# 101 A 15-Year Review of Risk Factors and Outcomes of Burn Patients Leaving Against Medical Advice

**DOI:** 10.1093/jbcr/irad045.074

**Published:** 2023-05-15

**Authors:** Lauren Powell, Alexis Knutson, Melanie McCormick, Alexandra Lacey

**Affiliations:** University of Minnesota, Minneapolis, Minnesota; University of Minnesota, Minneapolis, Minnesota; University of Minnesota, Minneapolis, Minnesota; HealthPartners, Saint Paul, Minnesota; University of Minnesota, Minneapolis, Minnesota; University of Minnesota, Minneapolis, Minnesota; University of Minnesota, Minneapolis, Minnesota; HealthPartners, Saint Paul, Minnesota; University of Minnesota, Minneapolis, Minnesota; University of Minnesota, Minneapolis, Minnesota; University of Minnesota, Minneapolis, Minnesota; HealthPartners, Saint Paul, Minnesota; University of Minnesota, Minneapolis, Minnesota; University of Minnesota, Minneapolis, Minnesota; University of Minnesota, Minneapolis, Minnesota; HealthPartners, Saint Paul, Minnesota

## Abstract

**Introduction:**

Discharging against medical advice (AMA) can have significant, detrimental effects on burn patient outcomes. Prior studies in non-burn injured patients have found that patients who leave AMA have a higher risk for adverse 30-day and 12-month outcomes, as well as higher hospital readmission rates and healthcare expenditures, in comparison to patients who are discharged to home in a conventional manner. The goal of this study is to identify risk factors for burn patients who left AMA and suggest solutions to mitigate these factors.

**Methods:**

Data was collected at a Level 1 Trauma Center over a 15 year period (2007-2022). Patients who left AMA from the burn center were identified using discharge patient codes. Demographic information was collected, including: age, sex, past medical and substance use history, insurance status, and language barriers. Additional information was recorded, including: time to presentation, mechanism of injury, total body surface area (TBSA) burned, burn depth, number of ICU days, length of stay, treatment, consultant teams, readmission and/or known complications after leaving the hospital. Descriptive statistics were used to determine potential trends within this patient population.

**Results:**

Over 2007-2022, 37 patients were identified as having left AMA from the burn unit. The average patient age was 37-years-old. Of those who left AMA, 64.9% were male and 70.2% were identified as having a substance abuse history. The majority (51.4%) had Medicaid or State health insurance, 29.7% had no insurance, and 18.9% had private insurance. In regards to the type of injury, 43.2% presented due to a frostbite injury, with the majority of AMA patients sustaining a < 1% TBSA burn. Most (83.7%) had social work and/or case management involved during their admission and all (100%) had their involvement if the length of admission was greater than one day. Almost half (48.6%) returned to the ED within 2 weeks with complications relating to pain, need for dressing change, or infection.

**Conclusions:**

Leaving AMA is associated with greater patient morbidity, including higher rates of hospital readmission and subsequently, higher healthcare expenditures. This study found that burn patients leaving AMA were more likely to suffer from small (< 1% TBSA) burns, often related to frostbite injuries. These patients were more likely to have substance abuse, a psychiatric history, and the majority had Medicaid or state health insurance. Recruiting interdisciplinary care members, including social work, psychiatry, and addiction medicine, early may help these patients by encouraging completion of their hospital care and setting up crucial follow-up care.

**Applicability of Research to Practice:**

Recruiting interdisciplinary team members to meet with patients at higher risk for leaving AMA, including those with small burns, substance use history, or psychiatric diagnoses may be useful to help lower the incidence of AMA discharges within the burn unit.

